# Seasonality, Rather than Nutrient Addition or Vegetation Types, Influenced Short-Term Temperature Sensitivity of Soil Organic Carbon Decomposition

**DOI:** 10.1371/journal.pone.0153415

**Published:** 2016-04-12

**Authors:** Yu-Qi Qian, Feng-Peng He, Wei Wang

**Affiliations:** 1 Department of Ecology, College of Urban and Environmental Sciences, and Key Laboratory for Earth Surface Processes of the Ministry of Education, Peking University, Beijing, 100871, China; 2 Shenzhen Graduate School, Peking University, Shenzhen 518055, China; Institute for Sustainable Plant Protection, C.N.R., ITALY

## Abstract

The response of microbial respiration from soil organic carbon (SOC) decomposition to environmental changes plays a key role in predicting future trends of atmospheric CO_2_ concentration. However, it remains uncertain whether there is a universal trend in the response of microbial respiration to increased temperature and nutrient addition among different vegetation types. In this study, soils were sampled in spring, summer, autumn and winter from five dominant vegetation types, including pine, larch and birch forest, shrubland, and grassland, in the Saihanba area of northern China. Soil samples from each season were incubated at 1, 10, and 20°C for 5 to 7 days. Nitrogen (N; 0.035 mM as NH_4_NO_3_) and phosphorus (P; 0.03 mM as P_2_O_5_) were added to soil samples, and the responses of soil microbial respiration to increased temperature and nutrient addition were determined. We found a universal trend that soil microbial respiration increased with increased temperature regardless of sampling season or vegetation type. The temperature sensitivity (indicated by Q_10_, the increase in respiration rate with a 10°C increase in temperature) of microbial respiration was higher in spring and autumn than in summer and winter, irrespective of vegetation type. The Q_10_ was significantly positively correlated with microbial biomass and the fungal: bacterial ratio. Microbial respiration (or Q_10_) did not significantly respond to N or P addition. Our results suggest that short-term nutrient input might not change the SOC decomposition rate or its temperature sensitivity, whereas increased temperature might significantly enhance SOC decomposition in spring and autumn, compared with winter and summer.

## Introduction

The Earth’s mineral soils represent a large terrestrial reservoir of soil organic carbon (SOC) derived from the accumulation of detritus residues and by-products of microbial decomposition processes [1]. Soil carbon storage is larger than the sum of carbon storage in the atmosphere and plants, reaching 3300 Pg [2]. Taking into account rising global temperatures [3] and the large size of the SOC pool, if SOC decomposition by microbial activity is strongly temperature sensitive, even small increases in temperature may prompt a large release of C from soils, resulting in an increase in atmospheric CO_2_ and a positive feedback on future warming [3–6]. Although climate-carbon models suggest that warming will accelerate the release of CO_2_ from soils, the magnitude of this feedback is uncertain, mostly because of uncertainty in the temperature sensitivity of SOC decomposition [7–11].

Most simulation models of regional and global carbon cycles use a single, fixed, Q_10_ coefficient (defined as the increase in respiration rate per 10°C increase in temperature) to express the temperature sensitivity of SOC decomposition [12]. As decomposer microbes might differ in their ability or strategy to efficiently use SOC within different phases of the year [13–16], the temperature sensitivity of SOC decomposition might differ across different seasons [11]. Previous studies have demonstrated a distinct transition in the microbial community structure and function from winter to summer [17–19]. For instance, in the temperate area of north China, a shift in soil microbial community composition occurred with higher fungal: bacterial biomass ratio and gram negative (G-): gram positive (G+) bacterial biomass ratio in winter than in summer [19]. Fungi could release a large number of extracellular enzymes that can digest a wide variety of substrates, even complex organic compounds such as lignin [20, 21]. Different gram-staining groups of bacteria, categorized by their cell wall composition, were also found to have various substrate preferences and survival strategies [22]. Different microbial groups in different seasons have distinct optimal temperature ranges for growth and activity, and therefore increased temperature might differentially affect the response of the microbial community [23–26]. The feedback responses caused by altered temperature might also differ among different ecosystems types [6]. Nevertheless, it is still unclear whether a universal pattern in the seasonal change in temperature sensitivity of SOC decomposition exists among different ecosystems.

Apart from global warming, anthropogenic activities have greatly altered soil nitrogen (N) and phosphorus (P) availability in terrestrial ecosystems, making the prediction of soil microbial respiration and its temperature sensitivity more complicate. Nutrient addition, as a test of nutrient limitation and simulation of nutrient enrichment, has been extensively conducted in previous field fertilization experiments. N addition has been reported to commonly suppress soil microbial respiration in a series of *in situ* studies [27–29] and meta-analysis [30], probably due to a decrease in soil acidification, microbial biomass, extracellular enzyme activity and microbial N mining. N addition reduced Gram-positive bacterial biomass, which changed the microbial community composition and, furthermore, reduced the amount of enzymes produced by Gram-positive bacteria [31–33], indicating a possible potential decrease in temperature sensitivity of decomposition rates. P addition might result in a reduction in SOC decomposition via dropping the availability of C substrate for microbial growth or by forming a non-labile compound with P and metal oxides in soil [34, 35]. A research conducted on Alaskan arctic and boreal soils implies that the response of microbial respiration to N addition largely depends on soil type, temperature, and incubation time [36]. However, further experiment should be conducted to clarify the effect of nutrient addition on soil organic decomposition and its temperature sensitivity in different vegetation types.

We sampled soils in spring, summer, autumn, and winter from five vegetation types, including evergreen coniferous forest (*Pinus sylvestris* var. *mongolica*), deciduous coniferous forest (*Larix principis-rupprechtii*), deciduous broadleaved forest (*Betula platyphylla*), shrubland (*Malus baccata*), and grassland (*Leymus chinensis*) in Saihanba, Hebei Province, northern China, from May 2013 to July 2014. Soil samples were incubated at three temperatures (1, 10 and 20°C). N and P were added to soil samples, and soil microbial respiration was measured. Our objective was to investigate whether soil microbial respiration showed a universal response to increasing temperature and whether this trend was affected by sampling season, vegetation, or nutrient addition. Specifically, we expected that: (1) the temperature sensitivity of soil microbial respiration would be highest in winter and lowest in summer, due to the commonly reported negative correlation between soil temperature and Q_10_ [37]; and (2) N and P addition would suppress soil microbial respiration and its temperature sensitivity due to a decrease in soil acidification, microbial biomass, extracellular enzyme activity and microbial N mining with N addition [27–29] and a decrease in the availability of C substrate for microbial growth or a formation of a non-labile compound with P and metal oxides in soil with P addition [34, 35].

## Methods

The administration of the Saihanba Forestry Center gave permission for this research at each study site. We confirm that the field studies did not involve endangered or protected species. The study site was situated at Saihanba Forestry Center in Hebei Province, northern China (117°12′-117°30′E, 42°10′-42°50′N, 1400 m a.s.l.). The study site is characterized by semi-arid and semi-humid temperate climate, with long winters (November to March) and short summers. Climate records from 1964 to 2004 indicate an annual mean air temperature of −1.4°C and annual mean precipitation of 450.1 mm [6]. Soil in this area consists of Aeolian soil, meadow soil, and boggy soil [38].

The study site was within a typical forest-steppe ecotone in a temperate area of northern China. Primary forests were harvested via large-scale industrial logging in the late 1900s and have been replaced by secondary forests and plantations. This site contains the largest area of plantation forests in China, with dominant species of *P*. *sylvestris* var. *mongolica* (Mongolia pine, evergreen coniferous forest) and *L*. *principis-rupprechtii* (Prince Rupprecht’s larch, deciduous coniferous forest). The secondary forest mainly consists of *B*. *platyphylla* (birch, deciduous broadleaved forest). In addition, shrublands dominated by *M*. *baccata* (Siberian crabapple, shrubland) and meadow grassland (*L*. *chinensis*) are also very common. Wang et al. [6] provideed further details of the study site.

We sampled five local dominant vegetation types, including *P*. *sylvestris* var. *mongolica* (pine), *L*. *principis-rupprechtii* (larch), *B*. *platyphylla* (birch), *M*. *baccata* shrublands and *L*. *chinensis* grassland. The annual means of soil pH, moisture, bulk density, SOC and total nitrogen of the sampled sites are described (Table 1). Six 20 m × 20 m sampling plots were established in September 2012, in each of the five vegetation types, with 10 m buffer zones between each sampling plot. Topsoil (0–10 cm mineral soil) samples were collected from each of the sampling plots. Sampling was conducted in spring, summer, autumn, and winter (April 2013, July 2013, September 2013, and January 2014, respectively). Growing season soil was collected using a soil auger with a diameter of 5.8 cm, while frozen winter soil was collected with a shovel after clearing the snow cover (4–16 cm). Each soil used in the experiment was passed through a 2-mm sieve, thoroughly homogenized and then brought to the laboratory. Visible roots and stones were carefully removed prior to the incubation experiment. Data for winter grassland soils were not obtained.

**Table 1:** Soil physical and chemical characteristics at 0–10 cm depth (n = 6) of five sampled vegetation types.

	Pine forest	Larch forest	Birch forest	Shrubland	Grassland
Soil pH	6.34±0.09	5.94±0.09	5.92±0.16	6.2±0.12	5.77±0.16
Soil water content (% of dry weight)	0.12±0.09	0.13±0.08	0.21±0.07	0.13±0.11	0.12±0.08
Soil bulk density (g/cm^3^)	0.83±0.05	0.88±0.02	0.65±0.04	0.72±0.03	0.96±0.01
Soil organic carbon (mg/kg)	1.22±0.32	0.93±0.25	2.78±1.3	1.78±0.56	1.01±0.26
Soil total nitrogen content (%)	0.12±0.03	0.09±0.03	0.23±0.1	0.17±0.05	0.11±0.02

Values were means ± standard error.

Soils were kept in -70°C immediately after sampling for no more than 1 week, then were incubated in the laboratory under a factorial combination of temperature (3 levels: 1, 10 and 20°C) and nutrient addition (4 levels: no addition (water), nitrogen addition, phosphorus addition, and a combination of nitrogen and phosphorus addition). Each soil sample was divided into four subsamples for the nutrient addition experiment. Nitrogen (N) and phosphorus (P) were added as 0.035 mM NH_4_NO_3_ (equivalent to 5 g N per m^2^ or 0.35 mg N per g dry soil) and 0.03 mM P_2_O_5_ (equivalent to 5 g P per m^2^ or 0.36 mg P per g dry soil) [39]. Deionized water or nutrient solutions were added to control and treated soils, respectively, at a rate of 1 mL nutrient solution or water per 5 g fresh soil. Before starting the incubation, soil moisture was adjusted to 60% of water-holding capacity through the addition of deionized H_2_O. During the incubation period, soil moisture was kept stable by weighing and adjusted by adding deionized water. Soil microbial respiration rate was determined using the alkali absorption method [40]. Approximately 25 g of fresh soils were placed into 250 mL glass gas tight jars and incubated at 1, 10, and 20°C for 5–7 days. Respired CO_2_ was captured by a connecting vial with 5 mL 1 M NaOH and determined by titration with 0.25 M HCl. Because labile carbon is exhausted gradually with the increase in incubation time [23, 41, 42], we used the short-term response of microbial respiration to temperature and nutrient addition [43, 44].

The total microbial biomass and microbial community composition were determined using phospholipid fatty acids (PLFAs) analysis. The fatty acid 18:2ω6, 9 was recognized as the fungal biomarker [45]. Bacterial biomass was quantified as the sum of i14:0, i15:0, a15:0, 16:1ω9, 16:1ω7, i17:0, a17:0, cy17:0, 17:0, and cy19:0 [46]. We calculated the total lipids as an indicator of microbial biomass [47]. The details of the method can be seen in [19].

Multifactor analysis of variance (ANOVA) was used to examine the effects of sampling season, incubation temperature and nutrient addition on soil microbial respiration, followed by Tukey’s multiple comparison if significance was tested. Q_10_ was estimated by an exponential model of soil CO_2_ efflux and temperature (with least squares technique) as follows:

$$F=\beta_{0}e^{\beta_{t}T}$$ (1)

where F is soil CO_2_ efflux rate, T is soil temperature, and β_0_ and β_t_ are constants. The Q_10_ values were calculated as shown below:

$$Q_{10}=e^{10\times \beta_{t}}$$ (2)

Linear regression analysis was used to examine the relationship between the Q_10_ and the microbial biomass and fungal: bacterial ratios. Statistical analysis was performed using SPSS 18.0 for Windows (SPSS Inc., Chicago, IL, USA) and R software (version 0.98.490). Significant effects were determined at *P* < 0.05 unless otherwise stated.

## Results

### Effect of increased temperature on microbial respiration rate

**Fig 1:**
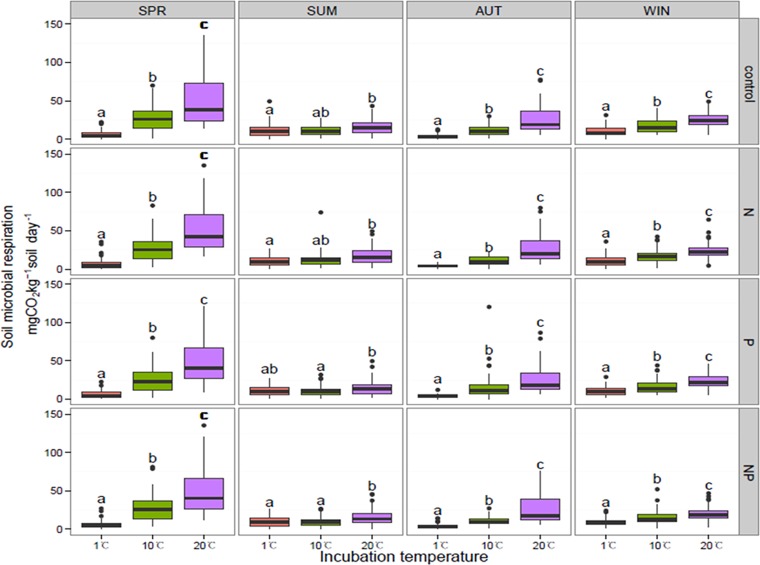
Comparison of soil microbial respiration at three incubation temperatures. SPR, SUM, AUT, and WIN represent spring, summer, autumn, and winter, respectively. Control, N, P, and NP represent different nutrient additions, including no nutrient addition but water; nitrogen addition, phosphorus addition, and a combination of nitrogen and phosphorus addition. Boxplot includes the data from five vegetation types. Significant differences among nutrient addition treatments are indicated by different letters. Values are means (n = 30) ± standard errors.

Microbial respiration rates were significantly different among 1, 10 and 20°C incubations (Fig 1), regardless of sampling time or vegetation type. Microbial respiration increased as temperature increased, and the soil microbial respiration rates at 1, 10 and 20°C were 6.34 ± 0.36 (mean ± s.e.), 26.32 ± 1.07, and 48.68 ± 1.85 mg CO_2_ kg^−1^ soil day^−1^ in spring soil; 10.69 ± 0.5, 11.13 ± 0.61, and 16.22 ± 0.68 mg CO_2_ kg^−1^ soil day^−1^ in summer soil; 3.96 ± 0.21, 12.14 ± 0.77 mg CO_2_ kg^−1^ soil day^−1^, and 26.83 ± 1.35 in autumn soil; 10.23 ± 0.5, 16.2 ± 0.66, and 23.31 ± 0.72 mg CO_2_ kg^−1^ soil day^−1^ in winter soil, respectively.

### Effect of sampling seasons on microbial respiration rates and Q10

**Fig 2:**
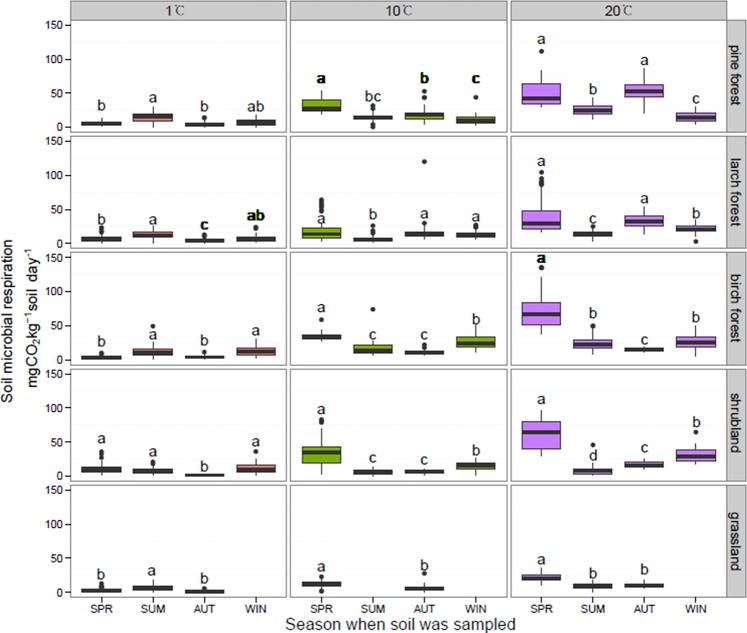
Comparison of soil microbial respiration among four seasons. Data are from all the nutrient treatment combinations. SPR, SUM, AUT, and WIN represent spring, summer, autumn, and winter, respectively. Significant differences among different sampling season are indicated by different letters. Values for winter and summer soil data incubated at 10°C in the grassland were absent because of accidents in sampling and experiments.

**Fig 3:**
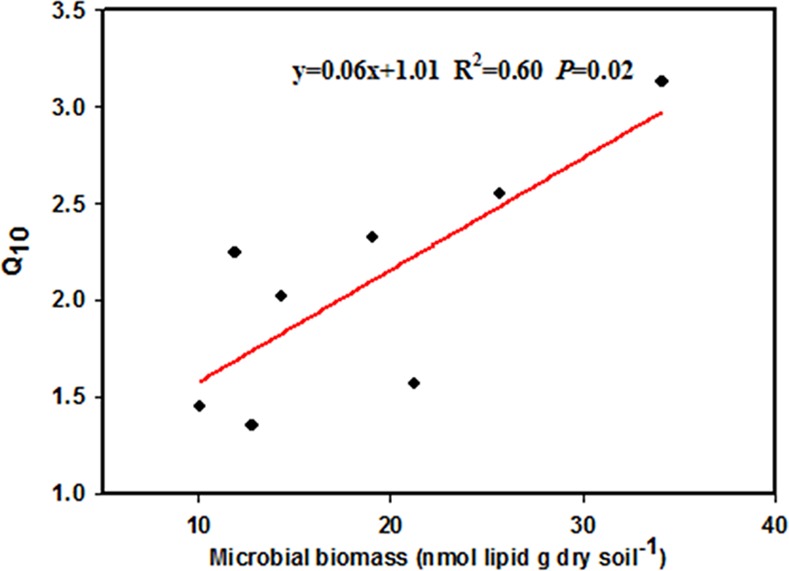
Relationship between Q_10_ of soil microbial respiration and microbial biomass. Data are from all the nutrient treatment combinations, vegetation types, and seasons.

**Fig 4:**
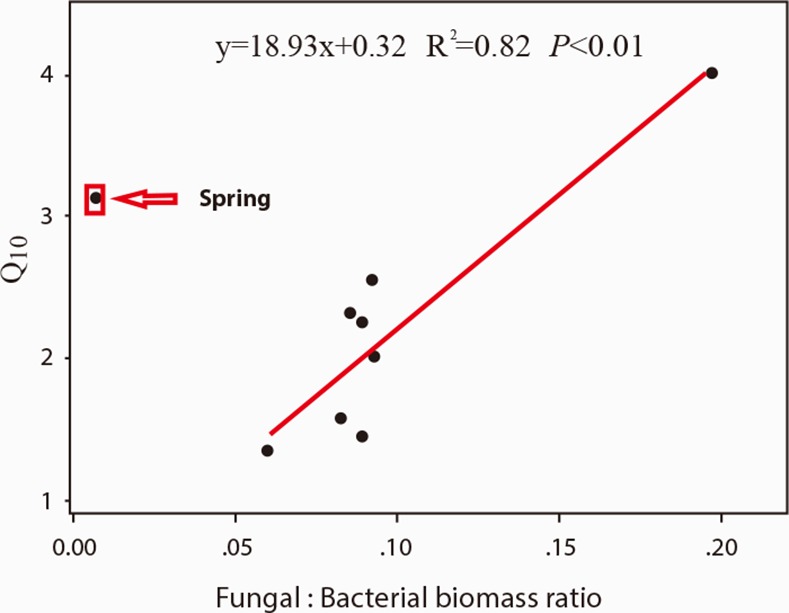
Relationship between Q_10_ of soil microbial respiration and fungal: bacterial biomass ratio. Data are from all the nutrient treatment combinations, vegetation types, and seasons.

When incubated at the same temperature, soil sampled from different seasons presented significantly different respiration rates (Fig 2). At 1°C, even though summer and winter soil had higher respiration rates than spring and autumn soil (*P* <0.05), the absolute respiration rates of all four seasons were very low. At 10 and 20°C, spring soil had a higher respiration rate than any other soil (*P* < 0.05). Temperature sensitivities of soil microbial respiration, expressed as Q_10_, were 3.13 (spring, R^2^ = 0.584, *P* < 0.001), 1.35 (summer, R^2^ = 0.076, *P* < 0.001), 2.78 (autumn, R^2^ = 0.521, *P* < 0.001), and 1.45 (winter, R^2^ = 0.179, *P* < 0.001). Soils from all of the five vegetation types showed higher Q_10_ in spring and autumn than in summer and winter. The seasonal pattern in Q_10_ was significantly correlated with microbial biomass (*Y* = 0.06 *X* + 1.01; R^2^ = 0.60, *P* = 0.02, Fig 3) and fungal: bacterial biomass ratio (*Y* = 18.93 *X* + 0.32; R^2^ = 0.82; *P* < 0.01, Fig 4).

### Effect of vegetation types on microbial respiration rates and Q10

**Fig 5:**
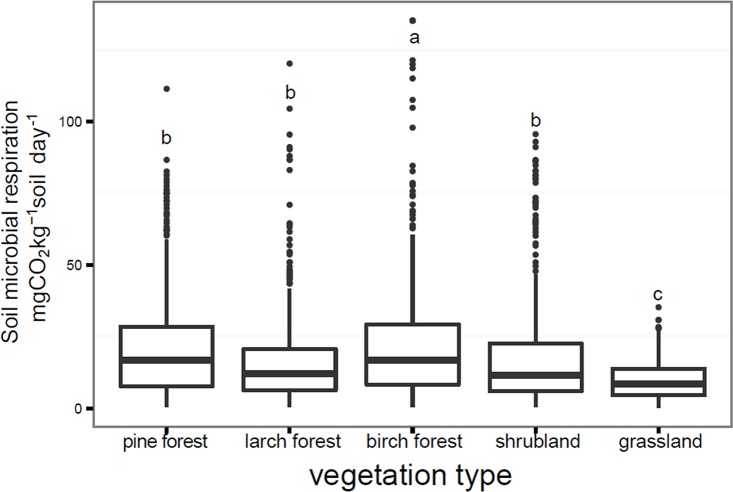
Comparison of soil microbial respiration among vegetation types. Data are from all the nutrient treatment combinations. Significant differences among different vegetation types are indicated by different letters. Samples from all seasons were included. Data are from all the nutrient treatment combinations.

Grassland soil had lower respiration rates than the other soils, while soil from the birch forest had the highest respiration rate except in autumn (Fig 5). The coefficients of variation of respiration rates were 0.85 in pine forest, 0.94 in larch forest, and 0.97 in birch forest, 1.03 in shrubland, and 0.71 in grassland. Q_10_ values of soil microbial respiration estimated in the five vegetation types were 2.33 (pine forest, R^2^ = 0.377), 2.02 (larch forest, R^2^ = 0.351), 2.55 (birch forest, R^2^ = 0.530), 2.25 (grassland, R^2^ = 0.372), and 1.57 (shrubland, R^2^ = 0.099). There was no significant difference in Q_10_ across vegetation types (*P* > 0.05).

### Effect of nutrient addition on microbial respiration rates and Q_10_

**Fig 6:**
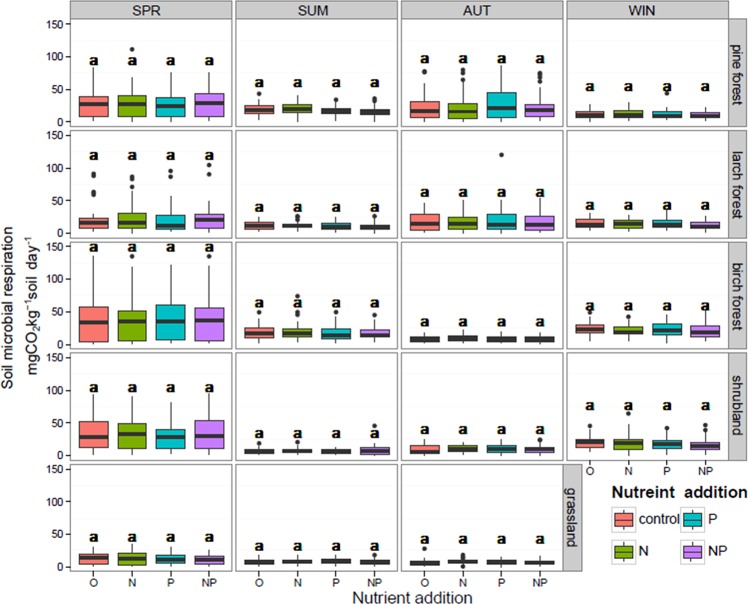
Effect of nutrient addition on soil microbial respiration rates. Data are from all temperature treatments. SPR, SUM, AUT, and WIN represent spring, summer, autumn, and winter. Significant differences among different nutrient addition treatments are indicated by different letters. Values from winter soil of grassland was absent because of accidents in sampling and experiments.

Mean soil microbial respiration rates among all the vegetation types were 18.61 ± 0.73, 18.93 ± 0.74, 18.11 ± 0.72 and 17.58 ± 0.74 mg CO_2_ kg^−1^ soil day^−1^ for control (water only), N addition, P addition, and combination of N and P addition treatments, respectively. For all soil samples, we did not find any significant response of soil microbial respiration to N, P or NP addition (Fig 6, multi-comparison, *P*>0.05). Using three-way ANOVA to determine the effects of nutrient addition, sampling season, and vegetation type, we did not find any significant response to nutrient addition, but significant responses occurred for sampling season and vegetation types (Table 2). No significant effect was observed for N or P addition on the temperature sensitivity of soil microbial respiration. Q_10_ values for control, N addition, P addition, and NP addition treatments were 2.08, 2.10, 1.97, and 2.01, respectively.

**Table 2:** Results of the two-way ANOVA used for detecting the effects of incubation temperature and nutrient addition on soil microbial respiration. Data are derived from all vegetation types.

Source of variation	Df	F-value	*P*
Incubation temperature	2	247.15	<0.001
Nutrient addition	2	0.82	0.482
Incubation temperature × Nutrient addition	9	0.23	0.99

## Discussion

### Seasonal dynamics of temperature sensitivity of SOC decomposition

In our study, Q_10_ values in spring and autumn were close to those recorded by Mo et al. [48] in a cool-temperate deciduous broad-leaved forest in Japan, but our winter Q_10_ was lower than in previous studies [48, 49]. An *in situ* field experiment conducted in a beech forest observed short-term Q_10_ values ranging between 0.9 and 38, with the lowest Q_10_ values in summer and the highest in winter. In summer Q_10_ averaged 4.3, whereas in winter mean Q_10_ was 16 [50]. Mikan et al. [49] observed a stronger response of microbial respiration to increased temperature in winter than in summer. The discrepancy between our results and the previous studies might be because of different incubation temperatures. Our incubation temperatures for winter soil ranged from 1 to 20°C, whereas previous studies used lower temperatures, such as −10 to −0.5°C [49]. At incubation temperatures below 0°C, thawing can lead to diffusion of organic molecules through water films and cell metabolism, and can also activate dormant microbes, resulting in rapid changes in the microbial population and thus C mineralization [50]. To avoid the changes in soil moisture caused by freezing and thawing at temperatures under 0°C, we set the lowest temperature as 1°C. Considering the differences between previous studies and our research, our short-term Q_10_ should not be directly compared with previous studies using different incubation temperatures.

Unexpectedly, soil sampled in spring and autumn had higher temperature sensitivities (Q_10_ values were 3.13 and 2.78 in spring and autumn, respectively) than in summer and winter (Q_10_ values were 1.35 and 1.45 in summer and winter, respectively). These results were consistent with the seasonal plasticity hypothesis, which states that the temperature sensitivity of microbial respiration varies distinctly across seasons, without a directional pattern in response to soil temperature [51]. Unlike our results, some experiments observed constant temperature sensitivity [52–55], while others found that temperature sensitivity of microbial respiration declined with increasing temperature [56, 57].

The higher Q_10_ in spring and autumn in our study could mainly be attributable to two reasons. First, higher microbial biomass in spring (unpublished data) indicates that increased efficiency of carbon usage promotes the biomass of microbial decomposers and increases the loss of soil carbon. Similarly, Allison et al 2010 [58] found that decline in microbial biomass and degradative enzymes can explain the observed attenuation of soil-carbon emissions in response to warming. Secondly, various decomposer microbes differ in their ability/strategy to efficiently use soil organic matter [59, 60, 61], so shifting within the community composition may affect decomposition rates. Higher fungi to bacteria ratio in autumn (unpublished data) could contribute to its higher Q_10_ of microbial respiration due to the fact that fungi could release a great number of extracellular enzymes which can digest a wide variety of substrates, even complex organic compound as lignin [62,63]. Fungi and extracellular enzymes catalyze the conversion of polymeric SOC to dissolved organic carbon (DOC), which is presumed to be the rate-limiting step in SOC decomposition [64, 65]. We also observed that Q_10_ increased with increased microbial biomass (Fig 3) and fungal: bacterial ratio (Fig 4). Our results therefore indicated that microbial physiology, including biomass and community composition, exerted a significant influence on the response of microbial respiration to increased temperature. Our results were similar to studies conducted in coniferous forests of the Rocky Mountains, where significant seasonal succession of microbial communities largely explained the seasonal patterns of soil respiration [66]. However, previous studies did not quantify the association between soil microbial respiration and microbial community composition. In this study we found for the first time that higher fungal: bacterial ratios led to larger responses of microbial respiration to increasing temperature, apart from spring soil samples (Fig 4). These results suggest that any changes in microbial community composition resulting from changing environmental conditions (including temperature, precipitation and nitrogen deposition) will significantly influence microbial carbon emission in response to temperature changes. This has important applications for predicting microbial activity responses to global changes.

In addition to microbial biomass and composition, substrate availability may affect SOC decomposition and temperature sensitivities in different seasons via influencing microbial composition, biomass, and metabolism [67]. For instance, bacteria are the first group to trap and metabolize most of the easily-available organics [68, 69]. Fungi commonly target recalcitrant substrates compared with bacteria [70]. A shift in microbial community composition along with different substrate quality across different seasons may subsequently change the enzyme production since that microbes with different preference will ultimately influence the SOC decomposition and its temperature sensitivity. However, we did not conduct measurements of substrate usage across different seasons. Future studies should be conducted on the seasonal dynamics of substrate usage for soil microbe in order to explore the mechanisms of seasonal differences in temperature sensitivity of soil microbial respiration.

### Response of microbial respiration and its temperature sensitivity to nutrient addition

There was no response of soil microbial respiration or Q_10_ values to N or P addition. There are limited studies addressing the effects of nutrient additions on soil microbial respiration [34–36, 70], or temperature sensitivity of soil microbial respiration [35]. Field studies found that N addition could lead to lower soil microbial respiration by changing microbial composition and reducing microbial N mining [28, 30, 71, 72, 73]. Temperature sensitivity of total soil respiration was not influenced by N fertilization in larch and ash plantations [74] but was enhanced in subtropical forest, crop field, temperate grassland and shrubland [30, 75, 76]. Field experiments are limited because they cannot isolate the response of soil microbes from the plant response to N addition. There are limited studies involving P addition [77, 78], and no universal conclusion was reached on the effect of P addition on soil respiration and temperature sensitivity. Iyamuremye and Dick [79] and Nziguheba et al. [80] found that microbes accumulated P as polyphosphates in cells rather than utilizing P in microbial growth or activity, while Malik et al. [35] reported that inorganic P addition enhanced microbial respiration in a laboratory incubation experiment. In our study, the fact that microbial respiration was not significantly influenced by nutrient addition indicated that soil microbes might not be limited by nutrients in the short term. A field experiment conducted in pine forest in our sampling area also found no response of microbial respiration to N or P addition (unpublished data). The lack of response might have also resulted from the low level of nutrient addition as well as the short incubation time; therefore, further research involving these two factors is needed to determine the possible nonlinear and long-term effects of nutrient addition on soil microbial respiration.

## Conclusions

Temperature sensitivity of soil microbial respiration is of increasing importance for its application in modeling and predicting terrestrial C flow in the context of global warming. Specifying the seasonal changes in temperature sensitivity of soil microbial respiration has improved our ability to build models with higher accuracy. By measuring the temperature sensitivity of soil microbial respiration rate among different vegetation types and seasons, our study found summer and winter soil had lower temperature sensitivity than spring and autumn soil, irrespective of vegetation types, indicating that microbial respiration had a distinct seasonal pattern in its response to increased temperature. Microbial biomass and fungal: bacterial biomass ratio significantly correlated with the seasonal pattern in the temperature sensitivity of microbial respiration. Our results suggest that any changes in microbial community composition resulting from changing environmental conditions (including temperature, precipitation and nitrogen deposition) will influence microbial carbon emission in response to temperature changes, which has important applications for predicting microbial activity responses to global change. In addition, soil microbial respiration and its temperature sensitivity were not influenced by nitrogen and phosphorus addition. Even though previous evidence suggested potential effects of nutrient addition on soil microbial respiration, contradictory results, including those obtained in this study, revealed that N and P availability might be a minor factor influencing carbon mineralization compared to increased temperature in the short-term.

## Supporting Information

S1 Appendix Respiration Season Vegetation Nutrient. Sample season SPR, SUM, AUT, and WIN represent spring, summer, autumn, and winter, respectively. Control, N, P, and NP represent different nutrient additions, including no nutrient addition but water; nitrogen addition, phosphorus addition, and a combination of nitrogen and phosphorus addition. Incubation Temperature 1, 10, 20 represent 1°C, 10°C, and 20°C. Unit of Respiration is mg CO_2_ kg^−1^ soil day^−1^.
(XLSX)

S2 Appendix Biomass FB ratio Q_10_. (XLSX)
